# Molecular epidemiological surveillance of circovirus in avian and mammalian species on the Brazilian coast

**DOI:** 10.1007/s00705-026-06659-5

**Published:** 2026-05-28

**Authors:** Ana Julia Chaves Gomes, Yasmin Luisa Neves Lemes Garcia, Dayla Bott Geraldini, Camila Domit, Fábio Henrique de Lima, João Pessoa Araújo, Juliana Schons Gularte, Micheli Filippi, Vyctoria Malayhka de Abreu Góes Pereira, Meriane Demoliner, Alexandre Sita, Fernando Rosado Spilki, Marília de Freitas Calmon, Paula Rahal, Vivaldo Gomes da Costa

**Affiliations:** 1 Genomics Laboratory, Department of Biology, São Paulo State University (UNESP), São José do Rio Preto, 15054-000 SP Brazil; 2 Ecology and Conservation Laboratory, Federal University of Paraná (UFPR), 83255-976 PR Pontal do Paraná, Brazil; 3 Veterinary Molecular Diagnostic Laboratory, Institute of Biotechnology, São Paulo State University (UNESP), Botucatu, 01049-010 SP Brazil; 4 Molecular Microbiology Laboratory, Feevale University, Novo Hamburgo, 93525-075 RS Brazil

## Abstract

Circoviruses (CVs) infect diverse mammals, birds, and freshwater fish and are linked to diseases causing major economic losses. Despite being well studied in psittacines, their circulation in other avian groups and mammals is poorly understood. We conducted molecular surveillance of CVs in migratory birds and mammals along the Paraná coast, southern Brazil. *Sterna hirundinacea* specimen tested positive by Nested-PCR for GullCV. In the GullCV-infected bird, histopathology revealed nonspecific lesions, including marked splenic lymphoid atrophy, cachexia-related changes, acute tubular necrosis, and generalized congestion. Sanger sequencing revealed 83% nucleotide identity with CV sequences previously reported in gulls from the United States, which was consistent with results obtained by NGS and Rep-based phylogenetic analysis. These findings contribute to the understanding of CV diversity, distribution, and host range.

**Supplementary Information:**

The online version contains supplementary material available at 10.1007/s00705-026-06659-5.

## Introduction

Avian species provide the natural reservoir for numerous viral species and therefore gene source for evolution, emergence and dissemination of novel viruses [1]. Their global distribution and annual long-distance migrations facilitate viral coevolution and interspecies transmission [1, 2]. In this context, avian circoviruses (CV) are distinguished by their ability to infect a wide range of avian species and are often associated with diseases that result in significant economic losses.

CVs belong to the family *Circoviridae*, which comprise the smallest known animal viruses capable of autonomous replication and encoding their own capsid protein [3]. This family is divided into two distinct genera: *Circovirus* and *Cyclovirus* [4]. This classification is based on differences by the position of the origin of replication relative to the coding regions and the length of the intergenic [3]. CVs are non-enveloped viruses characterized by circular single-stranded DNA genomes of approximately 2 kb in length [5]. Structurally, they exhibit icosahedral symmetry (T = 1) with an average diameter ranging from 15 to 25 nm [6]. Members of these genus typically encode at least two major open reading frames (ORFs): one encoding the replication-associated protein (Rep) and another encoding the capsid protein (Cp) [3]. The Rep protein is highly conserved among CV species and contains endonuclease and helicase domains involved in rolling circle replication (RCR) [3].

Although members of the genus *Circovirus* have been identified in a variety of mammals, birds, and freshwater fish, knowledge of their biology has been largely gathered from porcine circoviruses [7, 8]. In swine, infections with porcine circovirus types 1 through 4 (PCV1–PCV4) are associated with a broad spectrum of clinical conditions, including postweaning multisystemic wasting syndrome, porcine dermatitis and nephropathy syndrome, granulomatous enteritis, porcine respiratory disease complex, reproductive disorders, and acute pulmonary edema [9]. In avian hosts, CVs such as psittacine Beak and Feather Disease Virus (BFDV), goose circovirus (GoCV), and duck circovirus (DuCV) are known to cause immunosuppression, feather abnormalities, growth retardation, and increased susceptibility to secondary infections [4, 10].

Among these diverse avian groups, aquatic birds, especially migratory ones, represent an important ecological vector for the dispersion of these viruses, being capable of establishing new endemic foci at great distances from the original infection site [1, 11]. Brazil stands out globally for its remarkable avian diversity and serves as a major destination for millions of migratory birds arriving annually via different flyways [12, 13]. However, surveillance and monitoring of CV in wildlife in South America, are still limited when compared to other regions of the world. In this context, the present study aims to investigate the occurrence of CV in migratory birds and mammalian on the coast of the state of Paraná. Furthermore, by identifying potential new avian hosts, the study contributes to expanding knowledge on the diversity and distribution of CV in natural ecosystems and supports the enhancement of epidemiological surveillance strategies in wild bird populations.

## Materials and methods

Biological samples from birds and mammals were collected between April and August 2024 through the Santos Basin Beach Monitoring Project (PMP-BS) along the Paraná coast, southern Brazil. The Brazilian Institute of the Environment and Renewable Natural Resources (IBAMA) as a requirement for the environmental licensing of PETROBRAS (IBAMA Permit: ABIO 640/2015). Samples included swabs and tissues obtained from live animals and carcasses at different decomposition stages. The type of sample collected was determined by the species and its condition, which ranged from live individuals (score 1) to carcasses in advanced stages of decomposition: fresh (score 2), moderate decomposition (score 3), and advanced decomposition (score 4) [14]. Molecular analyses were conducted at the Genomics Laboratory, Department of Biology, São Paulo State University (UNESP).

Histopathological analyses were performed using hematoxylin–eosin staining. Depending on the degree of decomposition, a comprehensive set of organs and tissues was selected for analysis, including adrenal glands, spleen, brain (cerebrum and/or cerebellum), heart and great vessels, stomach, liver, gonads, small and large intestines, skeletal muscle, pancreas, skin, lungs, kidneys, and thyroid and parathyroid glands. Total DNA was performed using the UNIXTRACTOR DNA and RNA Extraction System (Uniscience, Osasco, SP, BRA).

The extracted material was subjected to Nested-PCR, consisting of two cycling steps in the Veriti Thermal Cycler (Applied Biosystems^®^, Waltham, MA, USA) using the GoTaq^®^ Master Mix kit (Promega, Madison, WI, USA) and generic primers for the Replicase gene [15]. The first round of PCR consisted of an initial denaturation at 95 °C for 5 min, followed by 45 cycles of denaturation at 94 °C for 30 s, annealing at 46 °C for 1 min, and extension at 72 °C for 1 min, with a final extension at 72 °C for 5 min. The second round was performed under the same conditions, except for an annealing temperature of 56 °C. Primer sequences used for amplification are described in [15].

The results were analyzed by 1.5% agarose gel electrophoresis with visualization achieved through ethidium bromide staining. Samples presenting a band of approximately 350 bp were considered positive. Positive and negative controls were included in all reactions, and a GeneRuler 1 kb DNA Ladder (Thermo Fisher Scientific Inc., Waltham, MA, USA) was used as a molecular size marker.

Positive amplicons were sequenced using the Sanger method. Sequencing reactions were prepared using the BigDye Terminator v3.1 Cycle Sequencing Kit (Applied Biosystems^®^, Waltham, MA, USA) and nPCR primers (R2 and F2). Capillary electrophoresis was performed on a Spectrum Compact CE System (Promega, Madison, WI, USA). The resulting sequences were analyzed using the BLASTn tool available at the GenBank^®^ database (https://blast.ncbi.nlm.nih.gov/).

To prepare genomic libraries and perform next-generation sequencing (NGS), the nucleic acid extraction for metagenomic analysis was performed using the MagMAX™ CORE Nucleic Acid Purification Kit (Applied Biosystems, Waltham, MA, USA), with automated processing on the KingFisher™ Duo Prime system (Thermo Fisher Scientific Inc., Waltham, MA, USA). First-strand complementary DNA (cDNA) synthesis was carried out using SuperScript™ IV Reverse Transcriptase, followed by second-strand synthesis using Platinum™ SuperFi II DNA Polymerase (Thermo Fisher Scientific Inc., Waltham, MA, USA), according to the manufacturers’ instructions.

Metagenomic libraries were prepared using the Illumina^®^ DNA Prep Kit (Illumina Inc., San Diego, CA, USA), following the manufacturer’s protocol. Sequencing was performed on the Illumina NextSeq 1000 platform using the NextSeq P1 reagent kit (300 cycles). Geneious Prime version 2022.2.1.1 was used for read mapping and consensus genome generation.

Raw sequencing data were demultiplexed and converted to FASTQ format. Reads were processed using the Chan Zuckerberg ID (CZ ID) platform, including adapter removal, quality trimming, and filtering, according to the platform’s default parameters. Host-derived sequences were removed, duplicate reads were eliminated, and subsampling was performed to limit the dataset to a maximum of 1 million reads for single-end data or 2 million reads for paired-end data. After quality control, 68.02% of reads passed filtering criteria, resulting in 1,344,218 reads retained for downstream analysis.

Taxonomic classification was conducted by aligning reads to the NCBI NT and NR databases using Minimap2 and DIAMOND. Contigs were assembled using SPAdes, and reads were remapped to contigs using Bowtie2. Final taxonomic assignments were refined by aligning contigs against custom nucleotide and protein databases using BLASTN and BLASTX.

Phylogenetic analysis was performed using nucleotide sequences from the present study along with 55 reference sequences. Sequence alignment was conducted using the MUSCLE algorithm implemented in Geneious software. A phylogenetic tree was constructed using the neighbor-joining method with the Tamura–Nei substitution model. The robustness of the inferred tree was evaluated by bootstrap analysis with 1,000 replicates. Further details of all the methodologies mentioned above can be found in Supplementary Materials and Methods.

## Results

A total of 311 biological samples were collected from 114 individual animals, comprising avian (*n* = 102) and mammalian species (*n* = 12) along the coast of Paraná during four-month period. Multiple samples were obtained from each animal, depending on sample availability and condition, resulting in a variety of sample types, choana (*n* = 101), cloaca (*n* = 100), encephalic (*n* = 85), Oropharyngeal (*n* = 11), anal (*n* = 9), nasopharyngeal (*n* = 1), rectal (*n* = 1), tracheal (*n* = 1) and pancreas (*n* = 2).

**Fig. 1:**
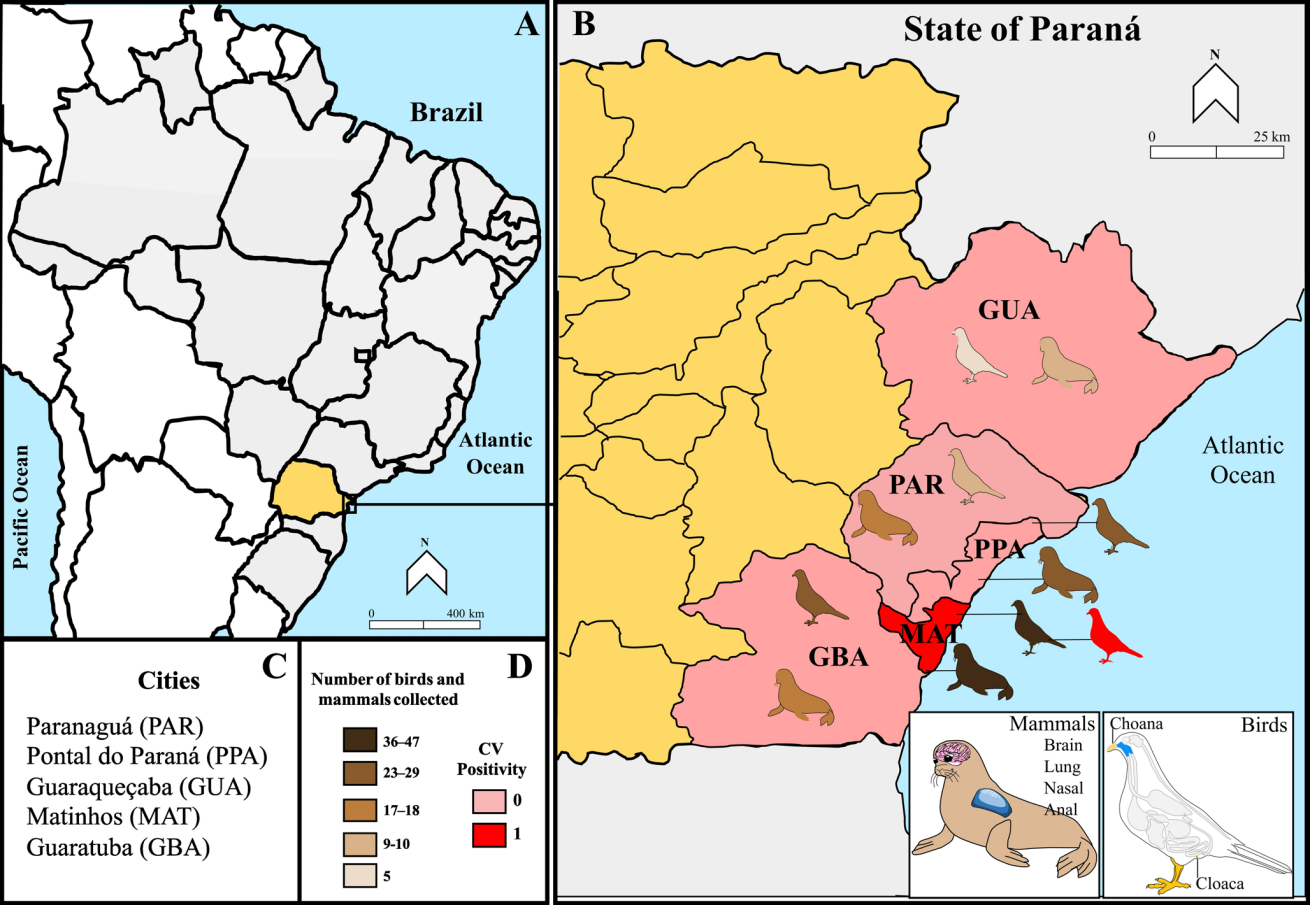
The geographical locations of the cities where the animals were collected are situated in coast of Paraná, Brazil (panel 1 A and 1B). Panel 1 C lists the 5 cities where the samples were collected in Paraná. Panel 1D displays colors indicating the number of animals collected by geographic region, with a bar representing the positive rate for CV

The animals were collected from 5 cities (Fig. 1) and proportions referring to animal-level data are presented using the total number of animals (*n* = 114) as the denominator. The largest number of animals collected occurred in Matinhos (51/114), followed by Paranaguá (21/114) and Pontal do Paraná (15/114), Guaratuba (18/114), and a smaller number in Guaraqueçaba (9/114).

Most individuals were juveniles (57/114), followed by adults (14/114) and neonates (1/114). The developmental stage could not be determined for 42 individuals (42/114) (Table S1).

A total of 19 bird species, distributed across 5 orders and 10 families, were identified, along with 4 mammal species belonging to 2 orders and 3 families (Table 1). The most frequently observed species were *Spheniscus magellanicus*, *Sula leucogaster*, and *Procellaria aequinoctialis*. Unfortunately, two species could not be identified, probably due to the advanced stage of decomposition.

**Table 1:** Distribution and relative abundance of avian and mammalian species on the coast of Paraná

Order	Family	Scientific name	No. of individuals
Procellariiformes	Procellariidae	*Calonectris sp.*	4
		*Puffinus gravis*	1
		*Procellaria aequinoctialis*	6
		*Puffinus griseus*	1
		*Fulmarus glacialoides*	1
		*Pachyptila sp.*	1
		*Macronectes giganteus*	1
	Diomedeidae	*Thalassarche melanophris*	1
Suliformes	Sulidae	*Sula leucogaster*	11
		*Sula dactylatra*	1
	Fregatidae	*Fregata magnificens*	2
	Phalacrocoracidae	*Phalacrocorax brasilianus*	4
Charadriiformes	Scolopacidae	*Calidris canutus*	3
	Laridae	*Thalasseus acuflavidus*	2
		*Larus dominicanus*	1
		*Sterna hirundinacea*	4
	Stercorariidae	*Stercorarius chilensis*	2
Sphenisciformes	Spheniscidae	*Spheniscus magellanicus*	53
Pelecaniformes	Threskiornithidae	*Phimosus infuscatus*	1
Carnivora	Otariidae	*Arctocephalus australis*	2
		*Arctocephalus tropicalis*	4
	Mustelídeos	*Lontra longicaudis*	1
Cetacea	Delphinidae	*Sotalia guianensis*	5
Total			112

* 2 unidentified individuals

The total refers to individual animals (*n* = 114). The number of samples (*n* = 311) is higher due to the number of samples per animal

Circovirus screening by Nested-PCR identified a single positive sample from a South American tern (*Sterna hirundinacea*) collected in Matinhos, Paraná State, Brazil. Among the analyzed swabs, CV DNA was detected exclusively in the choanal sample. Histopathological examination identified the animal as an immature male and revealed marked splenic lymphoid atrophy, lesions compatible with cachexia, acute tubular necrosis, and generalized congestion. Sanger sequencing confirmed the presence of CV DNA, showing 82.87% nucleotide identity with gull-associated CVs. Sequencing generated 9,885 reads and enabled the recovery of the complete Rep gene (912 nt).

**Fig. 2:**
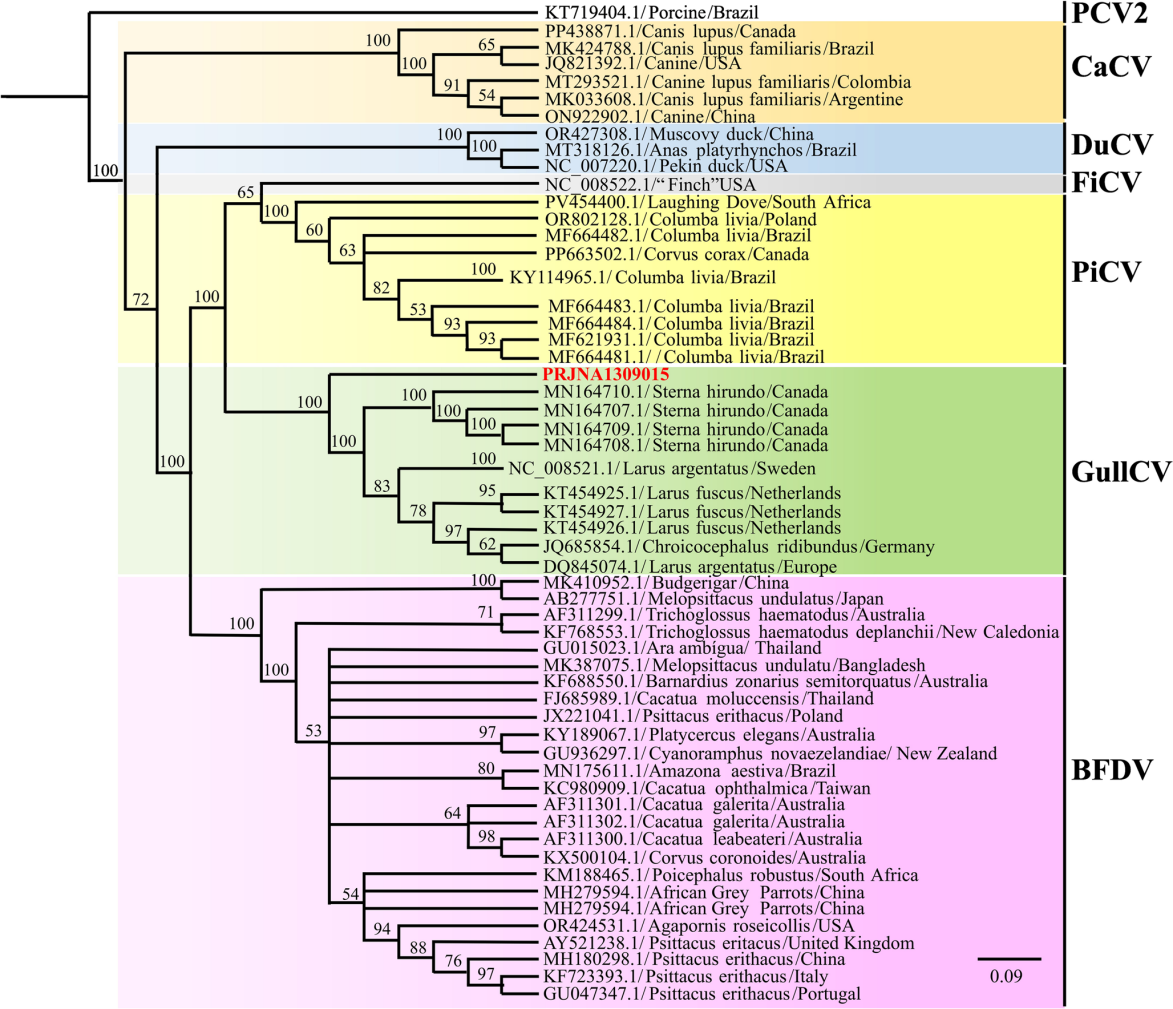
A phylogenetic tree based on in the nucleotide sequences of ORF1 (replicase gene) between the detected PRJNA1309015 (in red) and reference strains. Bootstrap values (> 50%) are shown at each node of the tree using 1,000 replicates. The scale indicates the number of divergent nucleotide residues. Sequences are labeled using GenBank accession numbers, species, and country where they were found. PCV2: Porcine circovirus type 2; CaCV: Canine circovirus; DuCV: Duck circovirus; FiCV: Finch circovirus; PiCV: Pigeon circovirus; GullCV: Gull circovirus; BFDV: Beak and feather disease virus

Phylogenetic analysis showed that the sequence obtained in this study clusters with sequences from Sterna Hirundo/Canada, forming a well-supported clade (bootstrap = 1000). Nucleotide BLAST (BLASTn) analysis indicated that the ORF1 (i.e., Rep gene) shares 83.86% nucleotide identity with the reference sequence MN164710.1/Sterna hirundo/Canada (Fig. 2).

## Discussion

The sequenced CV amplicon of the *Sterna hirundinacea* sample collected in the city of Matinhos showed similarity to sequences previously described in gulls in the United States. This genomic similarity may indicate a possible relationship among CVs infecting different avian species. Considering known migratory pattern to the Americas that connects bird populations between the Northern and Southern Hemispheres, this finding could be consistent with a potential role of migratory birds in the dispersal of viral lineages across regions [16].

Additionally, our results obtained through NGS indicated that it shares 83.86% nucleotide identity in the ORF1 (Rep) region with the reference sequence MN164710.1 (*Sterna hirundo*, Canada). This level of similarity indicates a genetic relationship with previously described CVs; however, it is based on a partial genomic region corresponding to a relatively conserved gene.

In addition, the absence of comparable CV sequences from Brazil in databases may have influenced this observation as it limits regional comparisons and could result in an apparent closer relationship with sequences from other geographic regions. Therefore, interpretations regarding the host range and geographic distribution should be made with caution, since these results are based on a single positive sample.

The role of CV and their association with the avian species investigated in this study remains poorly understood. To date, the most fully characterized avian CVs are those responsible for BFDV, which primarily affects birds of the order Psittaciformes, causing beak deformities, plumage abnormalities, and immunosuppression [17]. Despite this, studies reveal the importance of conducting further research to determine the infectivity and transmissibility of BFDV in non-psittacine species. The results suggest that all birds should be considered potential carriers of BFDV, regardless of species and clinical presentation [18, 19].

Among the few reports of possible CV infection in free-living birds, the detection of similar viral particles in a black-backed gull (*Larus dominicanus*) in New Zealand stands out, which presented signs of aspergillosis, the occurrence of which, according to histopathological analyses, suggested a possible association with immunosuppression related to lymphocytic depletion induced by CV [20]. Studies such as those conducted in zebra finches in southern Germany have reported lymphoid depletion in the spleen as a recurrent histopathological finding in birds with CV detection [21]. In this context, the histopathological alterations observed in the *Sterna hirundinacea* specimen analyzed in the present study were identified in the same individual in which CV DNA was detected. Although these findings are consistent with previous reports, they do not allow the establishment of a causal relationship between viral detection and the observed lesions. Therefore, histopathology should be considered a complementary tool for descriptive and comparative purposes, and further studies are required to clarify the potential role of CV in the development of such pathological changes.

In Brazil, studies on the circulation of CV, especially in migratory birds, are scarce, which limits the understanding of viral diversity, possible hosts, and ecological impacts associated with infection by these viruses. In this context, the evidence contained in this work points to the identification of a possible new host, suggesting that CV variants may pose a threat to populations of non-psittacine wild birds. Therefore, the identification of new potential hosts highlights the importance of surveillance in different avian groups, especially in migratory birds, which, when following intercontinental routes, can act as important vectors in the spread of the virus between different geographic regions and species [1, 16].

Although our study did not detect evidence of CV infection in the analyzed mammalian samples, recent research has reported the presence of CVs in these animals. One notable example is the detection of the Beaked Whale Circovirus (BWCV), an emerging viral agent identified in cetaceans [22, 23]. The presence of BWCV is particularly concerning, as its clinical and pathological relevance remains unclear, along with critical aspects such as host range, geographic distribution, prevalence, and pathogenic potential [24].

## Conclusion

In summary, this study contributes substantially to the understanding of CV circulation in migratory birds. The genomic similarity observed with sequences from gulls in the United States and Canada may be consistent with a potential role of migratory routes in the dispersal of viral lineages. Our findings also suggest that CV variants may also infect non-psittacine birds. Additionally, our findings suggest that CV variants may infect non-psittacine birds, indicating a potential new host. These results underscore the importance of active surveillance in wild bird populations and highlight the role of migration in the spread of avian pathogens.

## Supplementary Information

Below is the link to the electronic supplementary material.

Supplementary Material 1

## Data Availability

CV nucleotide sequences are available in the GenBank database under the accession numbers PV844622 (Sangersequencing), and PRJNA1309015 (NGS data).
