# Supplementary material for: Molecular epidemiological surveillance of circovirus in avian and mammalian species on the Brazilian coast
Source: Arch Virol. 2026 May 28;171(6):195. doi: 10.1007/s00705-026-06659-5 (PMC13219171; doi:10.1007/s00705-026-06659-5)
Supplement: Supplementary file 1 — Supplementary Material 1 [file 705_2026_6659_MOESM1_ESM.docx]

**SUPPLEMENTARY MATERIALS AND METHODS**

**1. DNA extraction**

DNA extraction was performed using the UNIXTRACTOR DNA and RNA Extraction System (Uniscience). The extraction process included the preparation of a lysis buffer composed of guanidine thiocyanate (Merck Millipore, Burlington, MA, USA, Ref. G9277) and N-lauroylsarcosine (Merck Millipore, Burlington, MA, USA, Ref. 61739), magnetic beads (Sera Mag™ SpeedBeads™ Carboxylate - Cytiva, Marlborough, MA, USA, Ref. GE44152105050350), proteinase K (Ludwig Biotecnologia Ltda., Porto Alegre, RS, Brazil) and absolute isopropanol (Merck Millipore, Burlington, MA, USA, Ref. 59304). All reagents were combined with the samples in a deepwell plate (Uniscience). The extracted material was subjected to washing steps using 80% DEPC Ethanol (Merck Millipore, Burlington, MA, USA, Ref. 51976) followed by elution of total DNA in Tris-HCl buffer (5 mM, pH 8), diluted in nuclease-free water.

**2. Nested-PCR**

The extracted material was subjected to Nested-PCR, consisting of two cycling steps in the Veriti Thermal Cycler (Applied Biosystems®) using the GoTaq® Master Mix kit (Promega, Madison, WI, USA, Ref. M7122) and generic primers for the Replicase gene. The first round of PCR included a 5 min incubation at 95 ^o^C, followed by 45 cycles, each consisting of 94 ^o^C for 30 s, 46 ^o^C for 1 min, and 72 ^o^C for 1 min, and a final incubation at 72 ^o^C for 5 min. The second round followed the same cycling conditions, except for the annealing temperature, which was 56 °C. The results were analyzed by electrophoresis on a 1.5% agarose gel prepared in TBE buffer and stained with ethidium bromide (10 mg/mL, 1%). The samples considered positive presented a 350bp band on the gel. The positive and negative controls used were internal and the marker used was the GeneRuler 1Kb DNA Ladder (Thermo Scientific^TM^, Vilnius, Lithuania, Ref. SM0311).

**3. Sanger sequencing**

**3.1 Amplicon DNA Purification**

Samples with amplicons of the expected size on agarose gel were purified again using magnetic beads (Cytiva, Marlborough, MA, USA, Ref 29729997). To 45 μL of PCR product, 90 μL of magnetic beads at room temperature were added and mixed by pipetting 10 times. The mixture was then incubated for 5 minutes, after which the tubes were placed on a magnetic rack and incubated for an additional 2 minutes. The supernatant was carefully removed without disturbing the beads, and 200 μL of 80% ethanol in water nuclease free was added for washing, followed by a 30-second incubation before ethanol removal. The wash step was repeated, and all residual ethanol was removed. The tubes were then left to air dry for 10 minutes at room temperature.

After drying, the tubes were removed from the magnetic rack, and 20 μL of DEPC-treated water was added. The mixture was homogenized by pipetting and incubated for 5 minutes. The tubes were then returned to the magnetic rack for 2 minutes, and the purified, eluted DNA was transferred to a new tube.

**3.2 BigDye Reaction**

This reaction was prepared using the BigDye Terminator v3.1 Cycle Sequencing Kit (Applied Biosystems, Foster City, CA, USA, Ref. 4337456), in duplicate and kept on ice throughout, using the following volumes: 2.5 μL of Milli-Q water, 1.5 μL of Save, 2 μL of BigDye, and 1 μL of N-PCR primers CV-F2 or CV-R2 (one mix for each replicate). Thermal cycling was performed under the following conditions: an initial step at 96 °C for 1 minute, followed by 35 cycles of 96 °C for 15 seconds, 50 °C for 15 seconds, and 60 °C for 4 minutes.

**3.3 DNA Precipitation**

The DNA precipitation protocol was performed as follows: initially, 2 µL of EDTA (125 mM) was added to the edges of the wells of a 96-well microplate containing the BigDye reaction products, and the plate was gently tapped to allow the droplets to settle at the bottom of the wells. Next, 2 µL of 3 M sodium acetate was added to the edges of the microplate, again followed by gentle agitation to facilitate sedimentation. Subsequently, 50 µL of 100% ethanol was added using a multichannel pipette, and the plate was vortexed at low speed.

The microplate was incubated at room temperature for 15 minutes, protected from light and covered with aluminum foil. After incubation, it was centrifuged at 4000 rpm for 30 minutes at 4 °C. Following centrifugation, the plate was inverted over a sink and placed on absorbent paper. While still inverted, it was centrifuged again for 1 minute at 1000 rpm at 4 °C to remove any residual liquid.

For the washing step, 70 µL of 70% ethanol was added using a multichannel pipette, followed by centrifugation for 15 minutes at 4000 rpm at 4 °C. The plate was again inverted over the sink and placed on absorbent paper, then centrifuged for 1 minute at 1000 rpm at 4 °C.

After this step, the plate was allowed to air dry for 20 minutes at room temperature. Once completely dry, it was for the subsequent sequencing step. Thus, 10 μL of formamide was added to each well, and the plate was heated at 95 °C for 1 minute. Subsequently, the plate was placed on ice for at least 2 minutes, and the precipitated fragments were sequenced using the automated DNA system Spectrum Compact CE System (Promega, Fitchburg, WI, USA), according to the manufacturer’s instructions.

**4. NGS library preparation**

Nucleic acid extraction was performed using the MagMAX™ CORE Nucleic Acid Purification Kit (Applied Biosystems, Waltham, MA, USA), and the extraction process was automated using the KingFisher™ Duo Prime system (Thermo Fisher Scientific Inc., Waltham, MA, USA). First-strand complementary DNA (cDNA) synthesis was performed using SuperScript™ IV Reverse Transcriptase (Thermo Fisher Scientific Inc., Waltham, MA, USA), followed by second-strand synthesis using Platinum™ SuperFi II DNA Polymerase (Thermo Fisher Scientific Inc., Waltham, MA, USA). All procedures were performed according to the manufacturers' protocols. Metagenomic libraries were subsequently prepared using the Illumina® DNA Prep Kit (Illumina Inc., San Diego, CA, USA), following the manufacturer's guidelines. Sequencing was performed on the Illumina NextSeq 1000 Sequencing System using the NextSeq P1 reagent kit (300 cycles) (Illumina Inc., San Diego, CA, USA).

Raw data were demultiplexed and extracted in the fastq format. The reads were treated to remove adapters and library indexes, trimming and filtering were performed previously with Chan Zuckerberg ID (CZ ID) software platform. Metagenomic analysis using the platform follows a flow divided into stages. Initially, data preprocessing occurs, which involves sending FASTQ files together with sample metadata. Next, file format validation is performed, adapters and low-quality or complex reads are removed, and host sequences are filtered. Duplicate reads are eliminated, and subsampling is applied to limit the number of sequences to a maximum of 1 million for single-end reads or 2 million for paired-end reads, to optimize computational performance.

In the species identification stage, sequences are first aligned to the NCBI NT and NR databases using the Minimap2 and DIAMOND tools. Subsequently, contigs are assembled using the SPAdes program, and reads are remapped to contigs using Bowtie2. The final phase of this step consists of aligning the contigs against custom nucleotide and protein databases using the BLASTN and BLASTX tools, enabling more accurate taxonomic identification

**5. Phylogenetic analysis**

The sample that tested positive for circovirus by NGS (i.e., Gull circovirus; GenBank accession no. PRJNA1309015), together with 55 additional representative sequences covering the ORF1 region (replicase gene) of circoviruses (see Figure 2 for GenBank accession numbers), was downloaded in FASTA format and imported into BioEdit [1]. The sequences were edited and aligned using the MUSCLE algorithm (Multiple Sequence Comparison by Log-Expectation) implemented in Geneious version 2022.2.1. Phylogenetic analysis was performed in the same software using the neighbor-joining method with the Tamura-Nei model. The robustness of the resulting tree was assessed by bootstrap analysis with 1,000 replicates, and nodes with bootstrap support >50% were considered reliable.

**Additional References**

1. Tom Hall (1999). BioEdit: a user-friendly biological sequence alignment editor and analysis program for Windows 95/98/NT. Nucleic Acids Symposium Series, 41, 95–98.

|  | | | | | | |
| --- | --- | --- | --- | --- | --- | --- |
| **Table S1.** List of analyzed specimens, including scientific name, biological samples collected, sampling location, developmental stage, stage of decomposition, and collection date | | | | | | |
| **Scientific Name** | **Biological samples** | | **Location** | **Developmental stage** | **Stage of decomposition** | **Collection date** |
| *Calonectris sp.* | | Choana, Cloaca and Encephalic | Matinhos | Adult | 2 | 25/04/2024 |
| *Calonectris sp.* | | Choana, Cloaca and Encephalic | Paranaguá | Adult | 2 | 28/04/2024 |
| *Puffinus gravis* | | Choana, Cloaca and Encephalic | Guaratuba | Juvenile | 2 | 29/04/2024 |
| *Sula leucogaster* | | Choana, Cloaca and Encephalic | Guaratuba | Adult | 2 | 30/04/2024 |
| *Calidris canutus* | | Choana and Cloaca | Guaratuba | Indeterminate | 1 | 03/05/2024 |
| *Calonectris sp.* | | Choana, Cloaca and Encephalic | Matinhos | Juvenile | 3 | 14/05/2024 |
| *Calidris canutus* | | Choana and Cloaca | Matinhos | Indeterminate | 1 | 15/05/2024 |
| *Calidris canutus* | | Choana and Cloaca | Matinhos | Indeterminate | 1 | 15/05/2024 |
| *Calonectris sp.* | | Choana, Cloaca and Encephalic | Matinhos | Juvenile | 3 | 15/05/2024 |
| *Thalassarche melanophris* | | Choana, Cloaca and Encephalic | Matinhos | Juvenile | 2 | 18/05/2024 |
| *Sotalia guianensis* | | Oropharyngeal, Anal, Encephalic and Nasopharyngeal | Guaraqueçaba | Juvenile | 5 | 20/05/2024 |
| *Thalasseus acuflavidus* | | Choana and Cloaca | Matinhos | Indeterminate | 1 | 28/05/2024 |
| *Stercorarius chilensis* | | Choana and Cloaca | Guaraqueçaba | Adult | 1 | 01/06/2024 |
| *-* | | Choana and Cloaca | Guaratuba | Indeterminate | - | 11/05/2024 |
| *-* | | Choana and Cloaca | Paranaguá | Indeterminate | - | 11/05/2024 |
| *Thalasseus acuflavidus* | | Choana, Cloaca and Encephalic | Guaraqueçaba | Juvenile | 2 | 19/08/2024 |
| *Sula leucogaster* | | Choana, Cloaca and Encephalic | Matinhos | Adult | 2 | 18/08/2024 |
| *Arctocephalus australis* | | Oropharyngeal, Anal and Encephalic | Paranaguá | Juvenile | 2 | 20/08/2024 |
| *Larus dominicanus* | | Choana, Cloaca and Encephalic | Matinhos | Adult | 2 | 20/08/2024 |
| *Sula leucogaster* | | Choana and Cloaca | Paranaguá | Indeterminate | 1 | 18/08/2024 |
| *Spheniscus magellanicus* | | Choana and Cloaca | Guaratuba | Juvenile | 1 | 19/08/2024 |
| *Spheniscus magellanicus* | | Choana and Cloaca | Paranaguá | Juvenile | 1 | 02/08/2024 |
| *Spheniscus magellanicus* | | Choana and Cloaca | Pontal Do Paraná | Indeterminate | 4 | 06/08/2024 |
| *Fregata magnificens* | | Choana and Cloaca | Matinhos | Adult | 1 | 03/08/2024 |
| *Spheniscus magellanicus* | | Choana and Cloaca | Matinhos | Juvenile | 2 | 05/08/2024 |
| *Phalacrocorax brasilianus* | | Choana and Cloaca | Pontal Do Paraná | Indeterminate | 1 | 02/08/2024 |
| *Sula leucogaster* | | Choana, Cloaca and Encephalic | Paranaguá | Juvenile | 2 | 02/08/2024 |
| *Spheniscus magellanicus* | | Choana, Cloaca and Encephalic | Matinhos | Juvenile | 2 | 01/08/2024 |
| *Spheniscus magellanicus* | | Choana and Cloaca | Guaraqueçaba | Indeterminate | 1 | 04/08/2024 |
| *Sula leucogaster* | | Choana and Cloaca | Matinhos | Adult | 1 | 03/08/2024 |
| *Arctocephalus tropicalis* | | Oropharyngeal and Encephalic | Pontal Do Paraná | Indeterminate | 4 | 31/07/2024 |
| *Spheniscus magellanicus* | | Choana and Cloaca | Guaraqueçaba | Juvenile | 1 | 01/08/2024 |
| *Arctocephalus tropicalis* | | Trachea and pancreas | Matinhos | Juvenile | 4 | 08/08/2024 |
| *Phalacrocorax brasilianus* | | Choana and Cloaca | Guaratuba | Indeterminate | 1 | 05/08/2024 |
| *Procellaria aequinoctialis* | | Choana and Cloaca | Pontal Do Paraná | Indeterminate | 4 | 06/08/2024 |
| *Spheniscus magellanicus* | | Choana, Cloaca and Encephalic | Matinhos | Juvenile | 3 | 08/08/2024 |
| *Spheniscus magellanicus* | | Choana, Cloaca and Encephalic | Pontal Do Paraná | Adult | 3 | 02/08/2024 |
| *Phalacrocorax brasilianus* | | Choana, Cloaca and Encephalic | Guaratuba | Juvenile | 2 | 01/08/2024 |
| *Arctocephalus tropicalis* | | Oropharyngeal, Anal and Encephalic | Paranaguá | Indeterminate | 4 | 31/07/2024 |
| *Spheniscus magellanicus* | | Choana, Cloaca and Encephalic | Pontal Do Paraná | Juvenile | 3 | 06/08/2024 |
| *Arctocephalus australis* | | Oropharyngeal, Anal and Encephalic | Guaraqueçaba | Juvenile | 4 | 31/07/2024 |
| *Procellaria aequinoctialis* | | Choana, Cloaca and Encephalic | Guaratuba | Indeterminate | 2 | 05/08/2024 |
| *Procellaria aequinoctialis* | | Choana, Cloaca and Encephalic | Matinhos | Juvenile | 2 | 05/08/2024 |
| *Sterna hirundinacea* | | Choana, Cloaca and Encephalic | Guaratuba | Adult | 3 | 02/08/2024 |
| *Arctocephalus tropicalis* | | Oropharyngeal, Pancreas and Encephalic | Matinhos | Juvenile | 4 | 01/08/2024 |
| *Procellaria aequinoctialis* | | Choana, Cloaca and Encephalic | Matinhos | Juvenile | 3 | 02/08/2024 |
| *Sterna hirundinacea* | | Choana, Cloaca and Encephalic | Matinhos | Juvenile | 3 | 02/08/2024 |
| *Spheniscus magellanicus* | | Choana, Cloaca and Encephalic | Matinhos | Indeterminate | 3 | 04/08/2024 |
| *Spheniscus magellanicus* | | Choana, Cloaca and Encephalic | Pontal Do Paraná | Indeterminate | 2 | 18/07/2024 |
| *Sterna hirundinacea* | | Choana, Cloaca and Encephalic | Matinhos | Indeterminate | 4 | 29/07/2024 |
| *Spheniscus magellanicus* | | Choana, Cloaca and Encephalic | Guaratuba | Juvenile | 2 | 29/07/2024 |
| *Sotalia guianensis* | | Oropharyngeal, Anal and Encephalic | Paranaguá | Neonates | 4 | 24/07/2024 |
| *Sula leucogaster* | | Choana, Cloaca and Encephalic | Matinhos | Juvenile | 3 | 30/07/2024 |
| *Procellaria aequinoctialis* | | Choana, Rectal and Encephalic | Paranaguá | Indeterminate | 2 | 30/07/2024 |
| *Spheniscus magellanicus* | | Choana, Cloaca and Encephalic | Matinhos | Juvenile | 3 | 05/07/2024 |
| *Spheniscus magellanicus* | | Choana, Cloaca and Encephalic | Matinhos | Juvenile | 3 | 25/07/2024 |
| *Sotalia guianensis* | | Oropharyngeal, Anal and Encephalic | Matinhos | Juvenile | 3 | 30/07/2024 |
| *Sula leucogaster* | | Choana, Cloaca and Encephalic | Guaraqueçaba | Adult | 2 | 27/07/2024 |
| *Spheniscus magellanicus* | | Choana, Cloaca and Encephalic | Matinhos | Juvenile | 3 | 29/07/2024 |
| *Spheniscus magellanicus* | | Choana, Cloaca and Encephalic | Matinhos | Juvenile | 2 | 29/07/2024 |
| *Procellaria aequinoctialis* | | Choana, Cloaca and Encephalic | Matinhos | Juvenile | 2 | 30/07/2024 |
| *Spheniscus magellanicus* | | Choana and Cloaca | Matinhos | Juvenile | 1 | 23/07/2024 |
| *Spheniscus magellanicus* | | Choana, Cloaca and Encephalic | Guaratuba | Juvenile | 3 | 25/07/2024 |
| *Spheniscus magellanicus* | | Choana, Cloaca and Encephalic | Matinhos | Juvenile | 3 | 29/07/2024 |
| *Spheniscus magellanicus* | | Choana, Cloaca and Encephalic | Guaraqueçaba | Juvenile | 2 | 27/07/2024 |
| *Spheniscus magellanicus* | | Choana, Cloaca and Encephalic | Paranaguá | Juvenile | 2 | 28/07/2024 |
| *Spheniscus magellanicus* | | Cloaca and Encephalic | Guaratuba | Juvenile | 3 | 30/07/2024 |
| *Spheniscus magellanicus* | | Choana, Cloaca and Encephalic | Matinhos | Juvenile | 3 | 27/07/2024 |
| *Sula leucogaster* | | Choana and Cloaca | Matinhos | Juvenile | 1 | 27/07/2024 |
| *Fregata magnificens* | | Choana, Cloaca and Encephalic | Matinhos | Indeterminate | 2 | 19/07/2024 |
| *Spheniscus magellanicus* | | Choana and Cloaca | Matinhos | Indeterminate | 1 | 03/07/2024 |
| *Spheniscus magellanicus* | | Choana and Cloaca | Matinhos | Juvenile | 3 | 28/06/2026 |
| *Spheniscus magellanicus* | | Choana and Cloaca | Pontal Do Paraná | Juvenile | 1 | 28/06/2026 |
| *Sula dactylatra* | | Choana and Cloaca | Matinhos | Indeterminate | 1 | 30/06/2024 |
| *Spheniscus magellanicus* | | Choana, Cloaca and Encephalic | Matinhos | Juvenile | 2 | 30/06/2024 |
| *Spheniscus magellanicus* | | Choana, Cloaca and Encephalic | Pontal Do Paraná | Indeterminate | 3 | 01/07/2024 |
| *Spheniscus magellanicus* | | Choana, Cloaca and Encephalic | Pontal Do Paraná | Indeterminate | 4 | 30/06/2024 |
| *Sterna hirundinacea* | | Choana, Cloaca and Encephalic | Paranaguá | Juvenile | 4 | 02/07/2024 |
| *Spheniscus magellanicus* | | Choana, Cloaca and Encephalic | Paranaguá | Indeterminate | 2 | 03/07/2024 |
| *Spheniscus magellanicus* | | Choana, Cloaca and Encephalic | Matinhos | Indeterminate | 3 | 04/07/2024 |
| *Puffinus griseus* | | Choana, Cloaca and Encephalic | Paranaguá | Indeterminate | 3 | 02/07/2024 |
| *Spheniscus magellanicus* | | Choana, Cloaca and Encephalic | Matinhos | Juvenile | 3 | 30/06/2024 |
| *Spheniscus magellanicus* | | Choana, Cloaca and Encephalic | Paranaguá | Indeterminate | 3 | 01/07/2024 |
| *Spheniscus magellanicus* | | Choana and Cloaca | Guaratuba | Juvenile | 1 | 30/06/2024 |
| *Pachyptila sp.* | | Choana, Cloaca and Encephalic | Pontal do Paraná | Indeterminate | 3 | 19/07/2024 |
| *Sotalia guianensis* | | Oropharyngeal, Anal and Encephalic | Paranaguá | Indeterminate | 2 | 02/07/2024 |
| *Spheniscus magellanicus* | | Choana, Cloaca and Encephalic | Paranaguá | Juvenile | 3 | 01/07/2024 |
| *Sotalia guianensis* | | Oropharyngeal, Anal and Encephalic | Matinhos | Indeterminate | 2 | 14/07/2024 |
| *Spheniscus magellanicus* | | Choana, Cloaca and Encephalic | Paranaguá | Indeterminate | 2 | 04/07/2024 |
| *Spheniscus magellanicus* | | Choana, Cloaca and Encephalic | Matinhos | Indeterminate | 2 | 30/06/2024 |
| *Sula leucogaster* | | Choana and Encephalic | Guaratuba | Indeterminate | 3 | 03/07/2024 |
| *Spheniscus magellanicus* | | Choana, Cloaca and Encephalic | Guaratuba | Juvenile | 2 | 24/07/2024 |
| *Spheniscus magellanicus* | | Choana and Cloaca | Paranaguá | Indeterminate | 4 | 20/07/2024 |
| *Spheniscus magellanicus* | | Choana, Cloaca and Encephalic | Matinhos | Juvenile | 2 | 20/07/2024 |
| *Spheniscus magellanicus* | | Choana, Cloaca and Encephalic | Paranaguá | Juvenile | 2 | 22/07/2024 |
| *Sula leucogaster* | | Choana, Cloaca and Encephalic | Guaraqueçaba | Juvenile | 3 | 23/07/2024 |
| *Spheniscus magellanicus* | | Choana, Cloaca and Encephalic | Matinhos | Juvenile | 3 | 23/07/2024 |
| *Spheniscus magellanicus* | | Choana, Cloaca and Encephalic | Matinhos | Juvenile | 2 | 24/07/2024 |
| *Spheniscus magellanicus* | | Choana and Cloaca | Matinhos | Juvenile | 1 | 24/07/2024 |
| *Spheniscus magellanicus* | | Choana, Cloaca and Encephalic | Guaratuba | Juvenile | 2 | 23/07/2024 |
| *Phimosus infuscatus* | | Choana, Cloaca and Encephalic | Paranaguá | Adult | 2 | 22/07/2024 |
| *Spheniscus magellanicus* | | Choana, Cloaca and Encephalic | Matinhos | Juvenile | 3 | 23/07/2024 |
| *Stercorarius chilensis* | | Choana, Cloaca and Encephalic | Matinhos | Adult | 2 | 13/07/2024 |
| *Lontra longicaudis* | | Oropharyngeal, Anal and Encephalic | Matinhos | Indeterminate | 2 | 08/07/2024 |
| *Spheniscus magellanicus* | | Choana, Cloaca and Encephalic | Paranaguá | Indeterminate | 2 | 06/07/2024 |
| *Spheniscus magellanicus* | | Choana, Cloaca and Encephalic | Pontal do Paraná | Indeterminate | 2 | 06/07/2024 |
| *Spheniscus magellanicus* | | Choana, Cloaca and Encephalic | Matinhos | Juvenile | 3 | 07/07/2024 |
| *Phalacrocorax brasilianus* | | Choana, Cloaca and Encephalic | Pontal Do Paraná | Juvenile | 2 | 07/07/2024 |
| *Fulmarus glacialoides* | | Choana, Cloaca, Encephalic | Matinhos | Indeterminate | 3 | 12/07/2024 |
| *Spheniscus magellanicus* | | Choana, Cloaca and Encephalic | Pontal do Paraná | Indeterminate | 3 | 07/07/2024 |
| *Sula leucogaster* | | Choana, Cloaca and Encephalic | Guaratuba | Adult | 2 | 11/07/2024 |
| *Spheniscus magellanicus* | | Choana, Cloaca and Encephalic | Matinhos | Juvenile | 3 | 07/07/2024 |
| *Macronectes giganteus* | | Choana, Cloaca and Encephalic | Pontal do Paraná | Indeterminate | 3 | 07/07/2024 |
| *Spheniscus magellanicus* | | Choana, Cloaca and Encephalic | Guaratuba | Indeterminate | 2 | 11/07/2024 |
